# ‘We need more support and doctors that understand the process of tapering …’: A content analysis of free‐text responses to a questionnaire on discontinuing long‐term benzodiazepine receptor agonist use

**DOI:** 10.1111/hex.13962

**Published:** 2024-01-07

**Authors:** Tom Lynch, Cristín Ryan, Christy Huff, D. E. Foster, Cathal Cadogan

**Affiliations:** ^1^ School of Pharmacy and Biomolecular Sciences Royal College of Surgeons Dublin Ireland; ^2^ School of Pharmacy and Pharmaceutical Sciences Trinity College Dublin Dublin Ireland; ^3^ Benzodiazepine Information Coalition Midvale Utah USA; ^4^ Benzodiazepine Action Work Group Colorado Consortium for Prescription Drug Abuse Prevention Aurora Colorado USA

**Keywords:** benzodiazepines, behaviour, discontinuation, questionnaire, Z‐drugs

## Abstract

**Background:**

Many individuals worldwide continue to take benzodiazepine receptor agonists (BZRAs) long term (≥3 months). The aim of this study was to conduct a content analysis of the views and experiences of discontinuing long‐term BZRA use as documented in the free‐text responses of respondents to an online questionnaire examining mediators of behaviour change relating to the discontinuation of long‐term BZRA use.

**Design:**

The questionnaire was disseminated via online BZRA support groups to community‐based adults with either current or previous experience of long‐term BZRA use. The four free‐text questions focused on (1) barriers and (2) facilitators to discontinuing BZRA use; (3) additional supports required to discontinue BZRA use; and (4) additional comments regarding BZRA use. Response data were analysed using summative content analysis.

**Results:**

The most commonly reported barrier to BZRA discontinuation related to the consequences of stopping the medication, including withdrawal symptoms and the possibility of return of the original symptoms. The most common facilitator that respondents reported would help them in discontinuing BZRA use was support, primarily from medical professionals. Many respondents reported having been harmed or negatively affected in some way because of BZRA use. Several respondents expressed regret over ever taking BZRAs and/or reported that, with the benefit of hindsight, they should never have taken BZRAs in the first instance.

**Conclusion:**

The findings highlight the range of barriers faced by those attempting BZRA discontinuation and the importance of additional supports. Holistic and person‐centred approaches are needed to support discontinuation of long‐term BZRA use that considers an individual's personal circumstances and wider social context.

**Patient or Public Contribution:**

‘Experts by experience’ with previous experience of long‐term BZRA use were involved in developing the questionnaire and writing the manuscript as collaborators. Individuals with lived experience of taking BZRAs completed the questionnaire.

## 1 INTRODUCTION

Benzodiazepine receptor agonists (BZRAs) have multiple indications (e.g., anxiety, insomnia) but are also associated with risks of physical dependence, withdrawal symptoms and patient harm (e.g., falls/fractures, cognitive impairment).^1^ Guidelines recommend restricting BZRA prescriptions to <2–4 weeks.^2^, ^3^ However, BZRAs continue to be commonly prescribed worldwide, oftentimes for long‐term use (>3 months).^4^, ^5^, ^6^ For example, over 30 million adults (12.6% of the population) in the US reported BZRA use in the past year^6^ and it has previously been estimated that long‐term use accounts for 30%–40% of BZRA prescribing.^7^

As part of a multiphase research project, a theory‐based intervention was developed to support discontinuation of long‐term BZRA use in primary care.^8^, ^9^ The SAFEGUARDING‐BZRAs (Supporting sAFE and GradUAl ReDuctIon of loNG‐term BenZodiazepine Receptor Agonist uSe) toolkit is underpinned by the theoretical domains framework (TDF), an integrated framework of 33 behaviour change theories that comprises domains that are considered mediators (i.e., barriers, facilitators) of behaviour change.^10^ The SAFEGUARDING‐BZRAs toolkit comprises 24 behaviour change techniques (BCTs) that map to individual domains within the TDF. The toolkit provides an extensive library of BCTs that aim to support the safe and gradual discontinuation of long‐term BZRA use among willing individuals. However, given the range of domains that component BCTs map to, further development is needed to assist with identifying priority domains that need to be targeted in designing a tailored BZRA discontinuation plan at an individual level.

To address this, a TDF‐based questionnaire was developed to examine mediators of behaviour change relating to the discontinuation of long‐term BZRA use. The questionnaire was distributed to members of the online BZRA community (i.e., online support groups for individuals experiencing BZRA‐related withdrawal and/or other adverse effects) as part of a validation exercise that is reported separately.^11^ Four free‐text questions were included in the questionnaire. The aim of this component of the questionnaire study was to conduct a content analysis of respondents' views and experiences of discontinuing long‐term BZRA use as documented in the free‐text responses.

## 2 METHODS

### 2.1 Questionnaire development and validation

The development and validation of the TDF‐based questionnaire, as well as its reporting, were based on the methods used in previous research^12^ and are reported separately.^11^ Briefly, a TDF‐based questionnaire to examine mediators of behaviour change relating to the discontinuation of long‐term BZRA use was developed based on previous studies.^12^, ^13^ The initial questionnaire was developed using the 14 domains of TDF version 2^10^: ‘Knowledge’, ‘Skills’, ‘Social/Professional Role and Identity’, ‘Beliefs about Capabilities’, ‘Optimism’, ‘Beliefs about Consequences’, ‘Reinforcement’, ‘Intentions’, ‘Goals’, ‘Memory, Attention and Decision Processes’, ‘Environmental Context and Resources’, ‘Social Influences’, ‘Emotions’ and ‘Behavioural Regulation’. Up to three questions in relation to each domain were included relating to barriers/facilitators to BZRA discontinuation, which resulted in 52 TDF‐based questions (Appendix S1). The research team included two experts by experience with previous experience of long‐term BZRA use (C. H., D. E. F.) who assisted with the development and piloting of the questionnaire. The validation exercise comprised two separate rounds. During Round 1, the initial version of the questionnaire was developed and disseminated (October 2020–January 2021) to the target sample (adults with either current or previous experience of using BZRAs on a long‐term basis; ≥3 months) via online support groups (outlined further below). During Round 2, the questionnaire was refined based on Round 1 analysis and redisseminated to the same target sample (November and December 2021). This paper focuses specifically on the analysis of the free‐text responses to Round 1. No free‐text response data were collected in Round 2. Ethical approval was granted by the RCSI Research Ethics Committee (reference number: REC202001015).

### 2.2 Sampling

Sampling took place through the following private Facebook groups: ‘Beating Benzos’, ‘Benzo Recovery’, ‘Benzo Warrior Community’ ‘Benzo Recovery and Existence’, ‘Positives while Healing from Benzodiazepines’, ‘Benzo Withdrawal Support Group’ and 'Benzodiazepine Withdrawal Support Group 1.0—Tapering & Withdrawal’. Each group was set up as a community for people currently taking BZRAs, including those who are undergoing gradual dosage reduction. The number of members of each group varied from between 1200 and 8000 members. The groups allow members to share their experiences of BZRA use and discontinuation and to provide advice and support to others. The groups are private and posts are not accessible to nonmembers. Membership to the groups is free and involves verification of the authenticity of an individual's Facebook account.

In order to meet the study eligibility criteria, respondents had to be aged ≥ 18 years; have either current or previous experience of using BZRAs on a long‐term basis (≥3 months); and be living in the community (i.e., not based in in‐patient treatment settings) at the time of completing the questionnaire. There were no exclusion criteria based on geographical location or the use of other medications.

### 2.3 Data collection

The questionnaire was made available via Alchemer®. A link to the questionnaire was posted on each Facebook page by one of the group's administrators. This link was then accessible on the ‘news feed’ of all members. The administrator ‘pinned’ the questionnaire to the group's homepage so that it was the first visible post on the group's page. Upon clicking the link to the questionnaire, group members were provided with information about the study. They were then asked to self‐declare that they met the above eligibility criteria and that they consented to participating in the study by completing the questionnaire, which was entirely anonymous. In completing the questionnaire, respondents were asked to rate their agreement with the TDF‐based questions using a seven‐point Likert scale. With the exception of the free‐text questions at the end of the questionnaire, all questions within each section of the questionnaire needed to be completed in order to proceed through the questionnaire.

The four free‐text questions were as follows:

1. What is the main barrier preventing you from stopping your BZRA use?
2. What would be the main thing that would help you to stop your BZRA use?
3. What forms of additional support would you need to help you stop your use of BZRAs?
4. Do you have any additional comments that you would like to add regarding long‐term BZRA use?

There was no word count limit on free‐text responses.

Guidance on sample sizes for questionnaire validation exercises recommends a minimum sample of five respondents per questionnaire item.^14^ Therefore, the minimum target sample for Round 1 questionnaire (52 items) was 260 respondents. The total sample size was calculated based on the number of individuals who clicked on the questionnaire, which was used as an indicator of the number of individuals who saw the questionnaire in the group. This provided a baseline sample size in order to calculate the response rate, which was based on the number of completed questionnaires. To maximise the response rate, reminders were posted into the groups with a link to the questionnaire at regular intervals.^15^

### 2.4 Data analysis

Responses from each of the four free‐text questions were collated in a Microsoft Excel spreadsheet and analysed separately by two researchers (T.L,C.C) using summative content analysis.^16^ The researchers familiarised themselves with the free‐text responses by reading and rereading them. During this process, the researchers noted their initial thoughts on the data, as well as potential patterns, potentially relevant coding ideas and early impressions of potential themes. Any blank cells within the spreadsheet or responses that contained no relevant information were marked as ‘not answered’. Using an inductive approach, labels for codes were then developed that encompassed the researchers' thoughts in relation to the data. These codes formed the basis of the initial coding scheme that was used to code the rest of the data. Coding was compared between the researchers and any disagreements were resolved through discussion. Once the entire data set had been coded, codes were then categorised based on potential relationships or links between codes. These categories were used to organise and group specific codes into themes.

Each open‐text question was analysed separately. Each response was only coded once per respondent to a particular code/theme. However, some respondents' responses contained multiple elements that were coded to separate codes and themes. Frequency counts were generated listing the number of responses relating to each code and theme. Due to considerable overlap in responses to the questions relating to facilitators and additional supports for BZRA discontinuation (i.e., open questions 2 and 3), the data were merged, and each response was only coded once per respondent to a particular code/theme.

## 3 RESULTS

The overall response rate to Round 1 was 46.2% (271/587). The majority of respondents were female (77%, *n* = 208), and the median age was 52 years (range: 19–81 years). Most respondents resided in the United States (69%, *n* = 185), Canada (8%, *n* = 23) or the United Kingdom (8%, *n* = 21), with the remainder of the respondents spread across Africa (1.8%, *n* = 5), Asia (4.1%, *n* = 11), Europe (4.1%, *n* = 11), Latin America (0.4%, *n* = 1) and Oceania (5.2%, *n* = 14). The majority of respondents were first prescribed BZRAs by either a general practitioner (45%, *n* = 118) or a psychiatrist (41%, *n* = 112). Almost half of the respondents (49%, *n* = 134) had been taking BZRAs for over 10 years, and the main reported reason for their initial BZRA prescription was anxiety (52%, *n* = 141). Most respondents (68%, *n* = 185) had previously attempted to discontinue BZRAs and 82% (*n* = 223) reported that they were tapering from BZRAs at the time of completing the questionnaire. Full details of Round 1 respondents' demographics and clinical characteristics are reported in Table 1. The number of respondents who submitted free‐text comments ranged from 179 to 216 across each of the four questions. Twenty‐four respondents did not respond to any of the free‐text questions.

**Table 1:** Respondent demographics and clinical characteristics.

	Frequency (*n* = 271; %)	
Gender	
Female	208 (76.8)	
Male	62 (22.8)	
Nonbinary	1 (0.4)	
Age (years)		
Median	52	
Range	19–81	
Country of residence		
Canada	22 (8)	
United Kingdom	22 (8)	
United States	187 (69)	
Other	41 (15)	
Highest level of education		
Bachelor's degree	76 (28)	
Master's degree	33 (12)	
Secondary school	122 (45)	
Other	41 (15)	
Employment status		
Full‐time employment	51 (19)	
Part‐time employment	22 (8)	
Retired	73 (27)	
Unemployed	73 (27)	
Other	51 (19)	
Relationship status		
Married	122 (45)	
Separated/divorced	49 (18)	
Single	65 (24)	
Widowed	8 (3)	
Other	27 (10)	
Clinical indication(s) for initial BZRA prescription^a^		
Anxiety	195 (70)	
Insomnia	72 (26)	
Muscle spasm	22 (8)	
Other	64 (23)	
Initial prescriber		
General practitioner	119 (44)	
Hospital doctor	16 (6)	
Psychiatrist	111 (41)	
Other	24 (9)	
Current BZRA(s)^a^		
Alprazolam	46 (17)	
Clonazepam	95 (35)	
Diazepam	70 (26)	
Lorazepam	62 (23)	
Other	62 (23)	
Number of BZRAs used		
1	228 (84)	
2	38 (14)	
≥3	5 (2)	
Duration of BZRA use (years)		
>10 years	133 (49)	
More than 5 years and less than 10 years	38 (14)	
More than 1 year and less than 5 years	68 (25)	
More than 3 months and less than 1 year	33 (12)	
Previously attempted BZRA discontinuation		
Yes	184 (68)	
Currently tapering from BZRA medication		
Yes	222 (82)	

Abbreviation: BZRA, benzodiazepine receptor agonist.

^a^ Some respondents selected >1 response options; therefore, values may not add up to 100%

### 3.1 Barriers to BZRA discontinuation

The most commonly reported barrier to BZRA discontinuation related to the consequences of stopping the medication (Theme: ‘Consequences of discontinuation’) (Table 2). Withdrawal symptoms and the possibility of return of their original symptoms (Theme: ‘Consequences of discontinuation’), as well as the associated fear that they instilled (Theme: ‘Fear’), were key causes of concern for respondents and acted as barriers to BZRA discontinuation.

I am tapering at 2.5% every two weeks from [lorazepam]. It seems as though once I get below a certain dose, the symptoms get very uncomfortable. (I have tried twice before). I am scared of the withdrawal symptoms. (PT_216)

…. the main barrier would be fear of anxiety returning. (PT_98)

**Table 2:** Overview of reported barriers to BZRA discontinuation (*n* = 197 respondents).

Theme	Subtheme	Frequency^a^ (*n*)
Consequences of discontinuation	Return of original symptoms	16
	Withdrawal symptoms	85
Fear	Fear of being without medication	3
	Fear of return of original symptoms	12
	Fear of withdrawal symptoms	32
	General fear/Fear of the unknown	4
Medical professionals	Lack of knowledge	8
	Lack of recognition of problems with BZRA use/discontinuation	5
	Lack of support	36
Personal circumstances	Caregiving responsibilities	9
	Dealing with existing health conditions	4
	Everyday family life	1
	Grief/bereavement	1
	Healthcare/insurance costs	2
	Lack of support/Living alone	11
	Work responsibilities/working while tapering	12
Tapering methods and resources	Identifying appropriate tapering methods	3
	Use of inappropriate tapering methods	5
	Lack of tapering resources and supports	10
	Tapering process	4
Other	Addiction/Dependence	3
	Time/Patience	2
	Necessity of the medication	3

Abbreviation: BZRA, benzodiazepine receptor agonist.

^a^ Frequency counts included as a general indicator of the number of individual responses relating to each subtheme.

A lack of support from medical professionals was another commonly reported barrier (Theme: ‘Medical professionals’).

Lack of support from the medical professionals. (PT_4)

I am currently tapering with no medical support … (PT_116)

Some respondents also reported a perceived lack of knowledge about how to prescribe and discontinue BZRAs among medical professionals, as well as a lack of recognition of the problems associated with BZRA use, including withdrawal symptoms (Theme: ‘Medical professionals’).

We need more support and doctors that understand the process of tapering. Not being told it is all in our heads. Coming off a benzo is very hard work and brings a lot of suffering if not done correctly. We need understanding and compassion. (PT_204)

Medical doctors are ignorant regarding weaning and side effects. (PT_218)

Respondents also reported various personal circumstances that acted as barriers to BZRA discontinuation (Theme: ‘Personal circumstances’). These included work and caregiving responsibilities, and the associated challenge of managing these while tapering.

Being a stay‐at‐home mom to multiple children, I feel I would need help with them to get off the benzodiazepines and I have no help. (PT_74)

Having to work while going through the withdrawal process. (PT_81)

The availability of tapering resources and supports was also noted as a barrier by a number of respondents (Theme: ‘Tapering methods and resources’)]. Some respondents reported challenges with identifying appropriate tapering methods, with a few respondents reporting the use of inappropriate tapering methods (e.g., abrupt discontinuation).

There are no resources readily available for tapering assistance. (PT_180)

Was previously cold turkeyed [abruptly withdrawn] from benzos and it was the most horrifying experience of my life, the thought of that happening again is a bit of a mental hurdle I have to overcome. (PT_239)

A number of respondents (*n* = 16) reported that there were no barriers to discontinuing BZRA use (Theme: ‘Other’). Some of these respondents qualified this by stating that they were already determined to stop the medication (Theme: ‘Other’).

I have no barriers. I'm determined to be off of them permanently. My mind is set on walking away from them. (PT_94)

### 3.2 Facilitators of BZRA discontinuation

The most common facilitator that respondents reported would help them in discontinuing BZRA use was support (Theme: ‘Support’) (Table 3). Respondents primarily referred to support from medical professionals.

Having the support of an educated doctor on benzodiazepines, and their help informing family that the withdrawal and damage is real and life altering. (PT_36)

**Table 3:** Overview of reported facilitators of BZRA discontinuation (*n* = 216 respondents).

Theme	Subtheme	Frequency^a^ (*n*)
Alternative treatment options	General/nonspecific	9
	Medication	24
	Natural approaches	2
	Therapy/counselling	31
Education	General/nonspecific	5
	Medical professionals	15
	Patients	19
Personal	Coping skills	12
	Determination/motivation	3
	Faith/hope	2
	Finances	20
	Improvements in life/health	15
Support	General/nonspecific	25
	Dedicated facility	25
	Employer	2
	Family/friends	38
	Medical professionals	111
	Online/peers	51
Tapering	Tapering plan	47
	Fewer withdrawal symptoms	13
Other	Time	1
	Research	3

Abbreviation: BZRA, benzodiazepine receptor agonist.

^a^ Frequency counts included as a general indicator of the number of individual responses relating to each subtheme.

Other reported forms of support included family and friends, dedicated facilities, as well as peers and online supports (Theme: ‘Support’).

Support and understanding from family. (PT_224)

Access to detox facilities for longer than 7 days. (PT_106)

Support online has helped me immensely. (PT_96)

A tapering plan was another common facilitator that respondents reported would help them in discontinuing BZRA use (Theme: ‘Tapering’).

If someone could come up with a good plan that I could follow (PT_80)

A proper regimen, protocol to slowly taper off. (PT_154)

Some respondents highlighted more specific requirements for tapering plans such as weighing scales, tablet crushers and water‐soluble tablets for achieving small dosage reductions (Theme: ‘Tapering’).

Having premade doses small enough to micro taper with. (PT_17)

Receiving a guide, and milligram scale and pill crusher. (PT_152)

Several respondents referred to the need for education for both the medical profession and patients regarding BZRA discontinuation (Theme: ‘Education’).

Education on how to taper safely. Not one of my doctors can tell me that. They think after 11 years I can taper every 3 days and be off in 2 weeks. (PT_10)

If the medical community was more well informed on the benzo withdrawal process and how long it takes to works your way off of a high dose. (PT_208)

Respondents also referred to the need for alternative treatment options, such as therapy/counselling, as well as therapeutic alternatives to address the symptoms for which BZRAs had initially been prescribed or to reduce withdrawal symptoms (Theme: ‘Alternative treatment options’).

Counsellor checking in with me a couple times a day. (PT_195)

If I could find another way to sleep without this medication. (PT_110)

A number of respondents also referred to changes in their personal circumstances and the development of coping skills as facilitators for discontinuing BZRA use (Theme: ‘Personal’)

Financial peace of mind. (PT_92)

How to cope with symptoms that come and go daily. Your mental health can be at stake daily. (PT_104)

Better living environment, more positive things in my life, a healthier diet and lifestyle (exercise, social life, etc.). (PT_56)

### 3.3 Additional comments regarding long‐term BZRA use

A range of views and experiences regarding long‐term BZRA use were included in the questionnaire's additional comments section (Table 4). Many respondents reported having been harmed or negatively affected in some way as a result of BZRA use (Theme: ‘Experience of BZRA use/discontinuation’).

The withdrawal makes you feel as though you have a chronic illness. The symptoms I have experienced are physical pain to the point if not wanting to get out of my bed. (PT_64)

They have ruined every aspect of my health and well‐being. (PT_70)

**Table 4:** Additional comments regarding long‐term BZRA use (*n* = 179 respondents).

Theme	Subtheme	Frequency^a^ (*n*)
Accountability	Pharmaceutical industry	4
	Prescribers	9
	Regulatory body	1
Advice for others	Avoid BZRA use	11
	Tapering advice/recommendations	6
Experience of BZRA use/discontinuation	Challenges of tapering/withdrawal	26
	Harmed by BZRAs	53
	Hindsight/regret ever taking BZRAs	25
	BZRAs provided benefit at one point	5
	Never educated/warned about potential harms/risks	47
Need for changes or restrictions to BZRA prescribing practices	Ban BZRAs	15
	Ban forced BZRA discontinuation	5
	Ban long‐term BZRA prescribing	10
	Need for informed consent	9
	Need for black box warnings	1
	Limit prescribing to specific prescribers	1
Prescriber education	Need for prescriber education	28
Recognition and support for BZRA users	Lack of recognition/support	22
	Need for dedicated tapering facilities	3
	Need for research	3
	Importance of online support groups	2

Abbreviation: BZRA, benzodiazepine receptor agonist.

^a^ Frequency counts included as a general indicator of the number of individual responses relating to each subtheme.

Several respondents expressed regret over ever taking BZRAs and/or reported that, with the benefit of hindsight, they should never have taken BZRAs in the first instance (Theme: ‘Experience of BZRA use/discontinuation’).

If I had one wish. Any wish, at all, it would be, I never filled that prescription. (PT_2250

A sizeable number of respondents also reported that they were never educated or warned about the potential harms and risks associated with the medication (Theme: ‘Experience of BZRA use/discontinuation’).

When I initially was prescribed [lorazepam], I didn't know what class of drug it was, or the danger of long‐term use. (PT_48)

I had no idea about the harm this medicine causes when I started taking it. I did not give informed consent. (PT_205)

Several respondents stated that there was a need for additional restrictions on BZRA prescribing [Theme: ‘Need for changes or restrictions to BZRA prescribing practices’]. Proposed restrictions included an outright ban on BZRAs, restrictions on quantity/duration of supply and the introduction of an informed consent process.

These drugs should be banned. (PT_122)

Informed consent needs to be mandatory before placing a patient on benzos. (PT_83)

It should be ILLEGAL for these to be prescribed longer than a month … (PT_105)

A number of respondents noted that forced discontinuation/withdrawal of BZRA use should also be banned (Theme: ‘Need for changes or restrictions to BZRA prescribing practices’), with some noting the importance for individuals to be supported to taper at their own pace (Theme: ‘Recognition and support for BZRA users’).

People should be allowed to taper at their own pace … For people who are already dependent upon them, they should not be forced to withdraw and there should be better supports in place from the medical community. (PT_55)

The laws definitely need to be changed as far as doctors with no knowledge of benzo withdrawal ripping you off improperly. (PT_178)

A number of respondents reported a need for prescriber education (Theme: ‘Prescriber education’) and for greater accountability in addressing the issue of long‐term BZRA use at the level of the prescribers, the pharmaceutical industry and regulatory bodies (Theme: ‘Accountability’).

Doctors need to be much more accountable and stop throwing them around like sweets. (PT_05)

The FDA [Food and Drug Administration] should be held accountable for what they have put us through due to this poison. (PT_37)

The availability by prescription, and the lack of warning is criminal! Big pharma should pay a price. (PT_1200

Some respondents outlined advice for others such as avoiding BZRA use, as well as recommendations on tapering (Theme: ‘Advice for others’).

My final advice would be to keep away from benzodiazepines. (PT_660

Don't do it. Do not take benzos. They cause long term & permanent damage to the brain & central nervous system. Once you take one, you have just opened the door to hell. (PT_213)

Take plenty of time and taper VERY small amounts. (PT_35)

A few respondents noted that the medication provided benefit at one point.

They truly helped me at one time. They really aren't the answer to my sleep issues which became worse post menopause. That is the only reason I went on them. Post‐menopausal symptoms were awful. (PT_940

## 4 DISCUSSION

This study reports on the analysis of free‐text responses to a questionnaire on barriers and facilitators to discontinuing long‐term BZRA use by members of the online BZRA community. Increasingly, individuals looking for support in discontinuing psychotropic medication are turning to online support groups and discussion forums and, as illustrated by the study findings, these platforms provide a novel and efficient means of engaging with them and learning from their experiences.^17^, ^18^, ^19^ In line with previous research,^8^, ^20^ the findings show that withdrawal symptoms and the possibility of the return of their original symptoms are common and persistent barriers to BZRA discontinuation. Both instilled fear in respondents, which acted as an additional barrier to BZRA discontinuation.

Another prominent barrier was a perceived lack of support from medical professionals, with several respondents stating that greater education on BZRAs, including withdrawal symptoms and tapering methods, was needed among medical professionals. This highlights an important mismatch between patient and prescriber experiences regarding BZRA discontinuation. Prescribers have previously reported encountering resistance from patients towards deprescribing BZRAs.^21^ This suggests that current efforts and resources to reduce long‐term BZRA use may not be effectively targeted at helping those who want to discontinue BZRA use. Looking at the wider literature on the discontinuation of psychotropic medication, individuals who have decided that they no longer want to continue taking psychotropic medication have reported encountering challenges in accessing professional support and having their autonomy and choice regarding treatment options respected.^22^ A previous qualitative study that explored mental health service users' experiences of discontinuing psychotropic medication reported mixed experiences of support from healthcare professionals with the process of medication discontinuation.^23^ Participants reported that they often had to develop ‘scaffolding strategies’ to navigate the process by themselves, which included the use of peer support. The importance of addressing this is further exemplified by the fact that the most common facilitator that respondents to the current questionnaire reported would help them in discontinuing BZRA use was support.

Respondents also reported various personal circumstances that acted as barriers to BZRA discontinuation. For example, several respondents discussed work and caregiving responsibilities, and the difficulty of managing these while tapering. These findings highlight some of the practical challenges faced by those who want to attempt to discontinue long‐term BZRA use. Previous qualitative research has highlighted that practical and emotional support from family members is an important factor that can influence an individual's motivation to discontinue BZRA use, but that family members may not always be aware that an individual is taking BZRAs or attempting discontinuation.^24^ These findings highlight the need for a holistic and person‐centred approach to supporting discontinuation of long‐term BZRA use that considers an individual's personal circumstances and wider social context.

The lack of availability of tapering resources and supports was also frequently noted as a barrier to discontinuing BZRA use. Respondents raised a wide range of issues from challenges with identifying appropriate tapering methods to the lack of dedicated tapering services. A recent global survey highlighted a paucity of existing deprescribing services for medications associated with dependence.^25^ Respondents to the current questionnaire also highlighted more specific requirements for tapering plans such as weighing scales, tablet crushers and water‐soluble tablets for achieving small dosage reductions. The topic of appropriate taper rates and durations for psychotropic medication is receiving considerable attention. Hyperbolic tapering regimens are now increasingly being recommended for discontinuing psychotropic medication in order to achieve linear reductions in pharmacological effect and mitigate against potential withdrawal symptoms.^26^, ^27^ These involve decreasing the dose of the medication based on fixed intervals of biological effect (e.g., 10% reduction in receptor occupancy) as opposed to decreasing the dose by fixed amounts. However, practical challenges exist to achieving increasingly smaller doses with existing marketed medications, particularly at the later stages of a hyperbolic tapering regimen. Some work has been undertaken to develop tapering strips consisting of psychotropic medication packaged into pouches of individual daily doses to enable gradual dosage reduction.^28^ However, these are currently not widely available or accessible on public health schemes, and their effectiveness requires further testing.^29^

In addition to reported barriers and facilitators to discontinuing long‐term BZRA use, respondents raised several other salient points about BZRA use in the questionnaire's additional comments section. A sizeable number of respondents reported having been harmed or negatively affected in some way as a result of BZRA use. A recent online survey highlighted that many individuals report experiencing multiple distressing symptoms because of BZRA use lasting from months to years after discontinuation.^30^ In light of the potential for protracted withdrawal symptoms resulting from BZRA use, a team of experts representing academic, clinical and lived experience of BZRA discontinuation has proposed the term ‘Benzodiazepine‐Induced Neurological Dysfunction’, which is defined as ‘a constellation of functionally limiting neurologic symptoms (both physical and psychological) that are the consequence of neuroadaptation and/or neurotoxicity to benzodiazepine exposure’.^31^ It is postulated that these symptoms may begin while taking or tapering BZRAs, and can persist following discontinuation for weeks, months or even years.

Several respondents also expressed regret over ever taking BZRAs and that, with the benefit of hindsight, they should never have taken BZRAs in the first instance. Previous studies examining patients' views and experiences of BZRA use have not reported such strong and emotive negative views regarding BZRA use.^20^ This may be reflective of the fact that respondents were sampled from private Facebook groups that act as dedicated sources of peer support to those currently taking BZRAs, including those who are undergoing gradual dosage reduction. A sizeable number of respondents also reported that they were never educated or warned about the potential harms and risks associated with the medication. This is consistent with the findings of a recent online questionnaire, whereby >75% of respondents stated that they had not been informed that benzodiazepines were intended for short‐term use only or that discontinuation can pose considerable challenges.^32^

Several respondents stated that there was a need for additional restrictions on BZRA prescribing, with some proposing an outright ban on BZRAs. Although such an approach may seem intuitive to reducing BZRA use and the associated potential for medication‐related harm, it could also have unintended consequences. For example, following a review of prescribed medications, such as BZRAs, that are associated with dependence and withdrawal, Public Health England warned that inappropriate restrictions could lead to harm, including an increased risk of suicide, and drive people to source medication from illicit or less‐regulated sources (e.g., the internet).^33^ It is, therefore, important that discontinuation strategies focus on supporting individuals to discontinue long‐term BZRA use safely using gradual dosage reduction and facilitating access to additional supports (e.g., counselling) wherever possible. A number of respondents also stated that forced discontinuation and withdrawal of BZRA use should also be banned, with some noting the importance for individuals to be supported to taper at their own pace. This is congruent with the approach recommended by the SAFEGUARDING‐BZRAs toolkit, whereby the co‐design team involved in its development do not encourage, or support, forced withdrawal or discontinuation of long‐term BZRA use.^9^ It also aligns with recent guidelines from the National Institute for Health and Care Excellence on safe prescribing of medicines associated with dependence or withdrawal symptoms, such as BZRAs.^34^ Finally, a small number of respondents noted that the medication provided benefit at one point. It is important not to lose sight of this as, despite the range of negative consequences that can arise with long‐term BZRA use, they can play an important role in various conditions when used in accordance with evidence‐based guidelines (e.g., as a short‐term measure during crises in individuals with anxiety). A persistent clinical challenge involves balancing the risks and benefits associated with BZRA use and ensuring that acute BZRA use does not become chronic.^35^

### 4.1 Strengths and limitations

This study's main strength is the extensive engagement with the online BZRA community, with a sizeable number of responses received across several different countries from individuals who had been prescribed the medication primarily by either general practitioners or psychiatrists. Given the prominent role of online communities as sources of information and support among those attempting psychotropic discontinuation,^17^, ^18^, ^19^ it is clear that much can be learned by engaging with individuals within these communities and learning from their experiences. In terms of study limitations, the questionnaire was disseminated through private Facebook groups, which introduced selection bias. Given that data collection overlapped with the earlier stages of the COVID‐19 pandemic, it would not have been practical to disseminate the questionnaire through clinical practice settings. Furthermore, as the questionnaire was disseminated through private Facebook groups and relied on voluntary completion, this may have introduced a self‐selection bias. For example, members of these communities may have had more negative experiences with the use of these medications, which could have influenced the findings, and there may be other demographic factors (e.g., country of residence, differences in healthcare system provision, socioeconomic factors) that influenced their experiences. However, given the prominence of these online communities, the questionnaire provided an efficient way of engaging with the target sample. It must also be noted that most questionnaire respondents were already tapering and this makes them distinct from individuals who are taking BZRAs on a long‐term basis and do not wish to discontinue the medication. However, engaging with individuals who actively want to discontinue long‐term BZRA use and learning from their experiences could help in developing interventions to support BZRA discontinuation. These interventions would then be available to support individuals who are currently not ready to change their existing behaviour if they decide to discontinue the medication in the future. Finally, due to the anonymous questionnaire format and reliance on text‐based data, it was not possible to seek additional clarification where responses were unclear or very short.

## 5 CONCLUSION

This study reports on a range of barriers faced by individuals in attempting BZRA discontinuation. The findings highlight the importance of additional supports from various sources by those attempting BZRA discontinuation, including medical professionals, family and friends, as well as peers and online supports. Holistic and person‐centred approaches are needed to support discontinuation of long‐term BZRA use that considers an individual's personal circumstances and wider social context.

## AUTHOR CONTRIBUTIONS

**Tom Lynch**: Conceptualisation; investigation; writing—original draft; methodology; formal analysis; project administration; writing—review and editing; funding acquisition. **Cristín Ryan**: Conceptualisation; investigation; funding acquisition; writing—review and editing; methodology; supervision; formal analysis. **Christy Huff**: Writing—review and editing; methodology; investigation. **D. E. Foster**: Investigation; methodology; writing— review and editing. **Cathal Cadogan**: Conceptualisation; investigation; funding acquisition; writing—original draft; methodology; writing—review and editing; supervision; formal analysis; project administration.

## CONFLICT OF INTEREST STATEMENT

The authors declare no conflict of interest.

## ETHICS STATEMENT

Ethical approval was granted by the RCSI Research Ethics Committee (reference number: REC202001015). All respondents self‐declared that they met the eligibility criteria and consented to completing the anonymous questionnaire.

## Supporting information

Supporting information.

## Data Availability

The data that support the findings of this study may be made available on reasonable request from the corresponding author. The data are not publicly available due to privacy or ethical restrictions.

## References

[1] Soyka M . Treatment of benzodiazepine dependence. N Engl J Med. 2017;376(12):1147‐1157. 28328330 10.1056/NEJMra1611832

[2] Pottie K , Thompson W , Davies S , et al. Deprescribing benzodiazepine receptor agonists: evidence‐based clinical practice guideline. Can Fam Physician. 2018;64(5):339‐351. 29760253 PMC5951648

[3] Kennedy KM , O'Riordan J . Prescribing benzodiazepines in general practice. Br J Gen Pract. 2019;69(680):152‐153. 30819759 10.3399/bjgp19X701753 PMC6400612

[4] Olfson M , King M , Schoenbaum M . Benzodiazepine use in the United States. JAMA Psychiatry. 2015;72(2):136‐142. 25517224 10.1001/jamapsychiatry.2014.1763

[5] Cadogan CA , Ryan C , Cahir C , Bradley CP , Bennett K . Benzodiazepine and Z‐drug prescribing in Ireland: analysis of national prescribing trends from 2005 to 2015. Br J Clin Pharmacol. 2018;84(6):1354‐1363. 29488252 10.1111/bcp.13570 PMC5980334

[6] Maust DT , Lin LA , Blow FC . Benzodiazepine use and misuse among adults in the United States. Psychiatr Serv. 2019;70(2):97‐106. 30554562 10.1176/appi.ps.201800321 PMC6358464

[7] Kurko TAT , Saastamoinen LK , Tähkäpää S , et al. Long‐term use of benzodiazepines: definitions, prevalence and usage patterns—a systematic review of register‐based studies. Eur Psychiatry. 2015;30(8):1037‐1047. 26545257 10.1016/j.eurpsy.2015.09.003

[8] Lynch T , Ryan C , Cadogan CA . ‘I just thought that it was such an impossible thing’: a qualitative study of barriers and facilitators to discontinuing long‐term use of benzodiazepine receptor agonists using the theoretical domains framework. Health Expect. 2022;25(1):355‐365. 34862703 10.1111/hex.13392 PMC8849267

[9] Lynch T , Ryan C , Bradley C , et al. Supporting safe and gradual reduction of long‐term benzodiazepine receptor agonist use: development of the SAFEGUARDING‐BZRAs toolkit using a codesign approach. Health Expect. 2022;25(4):1904‐1918. 35672924 10.1111/hex.13547 PMC9327818

[10] Cane J , O'Connor D , Michie S . Validation of the theoretical domains framework for use in behaviour change and implementation research. Implement Sci. 2012;7(1):37. 22530986 10.1186/1748-5908-7-37 PMC3483008

[11] Lynch T , Ryan C , Presseau J , et al. Development and validation of a theory‐based questionnaire examining barriers and facilitators to discontinuing long‐term benzodiazepine receptor agonist use. Res Social Adm Pharm. 2023;S1551-S7411(23):00467-9. 10.1016/j.sapharm.2023.10.015 37919219

[12] Seward K , Wolfenden L , Wiggers J , et al. Measuring implementation behaviour of menu guidelines in the childcare setting: confirmatory factor analysis of a theoretical domains framework questionnaire (TDFQ). Int J Behav Nutr Phys Act. 2017;14(1):45. 28376892 10.1186/s12966-017-0499-6 PMC5381057

[13] Taylor N , Lawton R , Conner M . Development and initial validation of the determinants of physical activity questionnaire. Int J Behav Nutr Phys Act. 2013;10(1):74. 23758912 10.1186/1479-5868-10-74 PMC3684519

[14] Kline RB . Principles and Practice of Structural Equation Modeling. 2nd ed. Guilford Press; 2005.

[15] Edwards PJ , Roberts I , Clarke MJ , et al. Methods to increase response to postal and electronic questionnaires. Cochrane Database Syst Rev. 2009;8(3):Mr000008. 10.1002/14651858.MR000008.pub4 PMC8941848 19588449

[16] Hsieh HF , Shannon SE . Three approaches to qualitative content analysis. Qual Health Res. 2005;15(9):1277‐1288. 16204405 10.1177/1049732305276687

[17] White E , Read J , Julo S . The role of Facebook groups in the management and raising of awareness of antidepressant withdrawal: is social media filling the void left by health services? Ther Adv Psychopharmacol. 2021;11:204512532098117. 10.1177/2045125320981174 PMC7816538 33520155

[18] Framer A . What I have learnt from helping thousands of people taper off antidepressants and other psychotropic medications. Ther Adv Psychopharmacol. 2021;11:204512532199127. 10.1177/2045125321991274 PMC7970174 33796265

[19] White E . Tapering antidepressants: why do tens of thousands turn to Facebook groups for support? Br J Gen Pract. 2021;71(708):315. 34319884 10.3399/bjgp21X716309 PMC8249018

[20] Sirdifield C , Chipchase SY , Owen S , Siriwardena AN . A systematic review and meta‐synthesis of patients' experiences and perceptions of seeking and using benzodiazepines and Z‐drugs: towards safer prescribing. Patient. 2017;10(1):1‐15. 27282559 10.1007/s40271-016-0182-z

[21] Niznik JD , Ferreri SP , Armistead LT , et al. Primary‐care prescribers' perspectives on deprescribing opioids and benzodiazepines in older adults. Drugs Aging. 2022;39(9):739‐748. 35896779 10.1007/s40266-022-00967-6 PMC9330848

[22] Keogh B , Murphy E , Doyle L , Sheaf G , Watts M , Higgins A . Mental health service users experiences of medication discontinuation: a systematic review of qualitative studies. J Ment Health. 2022;31(2):227‐238. 34126035 10.1080/09638237.2021.1922644

[23] Watts M , Murphy E , Keogh B , Downes C , Doyle L , Higgins A . Deciding to discontinue prescribed psychotropic medication: a qualitative study of service users' experiences. Int J Ment Health Nurs. 2021;30(1):1395‐1406. 34101332 10.1111/inm.12894

[24] Coteur K , Van Nuland M , Schoenmakers B , Van den Broeck K , Anthierens S . “At the time I only wanted to relieve stress”: exploring motivation for behaviour change in long‐term hypnotic users. Heliyon. 2023;9(5):e16215. 37234622 10.1016/j.heliyon.2023.e16215 PMC10205632

[25] Cooper RE , Ashman M , Lomani J , et al. “Stabilise‐reduce, stabilise‐reduce”: a survey of the common practices of deprescribing services and recommendations for future services. PLoS One. 2023;18(3):e0282988. 36920968 10.1371/journal.pone.0282988 PMC10016688

[26] Sørensen A , Ruhé HG , Munkholm K . The relationship between dose and serotonin transporter occupancy of antidepressants—a systematic review. Mol Psychiatry. 2022;27(1):192‐201. 34548628 10.1038/s41380-021-01285-w PMC8960396

[27] Horowitz MA , Taylor D . Tapering of SSRI treatment to mitigate withdrawal symptoms. Lancet Psychiatry. 2019;6(6):538‐546. 30850328 10.1016/S2215-0366(19)30032-X

[28] Groot PC , van Os J . Successful use of tapering strips for hyperbolic reduction of antidepressant dose: a cohort study. Ther Adv Psychopharmacol. 2021;11:204512532110393. 10.1177/20451253211039327 PMC8404667 34471516

[29] van Os J , Groot PC . Outcomes of hyperbolic tapering of antidepressants. Ther Adv Psychopharmacol. 2023;13:204512532311715. 10.1177/20451253231171518 PMC10185864 37200818

[30] Huff C , Finlayson AJR , Foster DE , Martin PR . Enduring neurological sequelae of benzodiazepine use: an Internet survey. Ther Adv Psychopharmacol. 2023;13:204512532211455. 10.1177/20451253221145561 PMC9905027 36760692

[31] Ritvo AD , Foster DE , Huff C , Finlayson AJR , Silvernail B , Martin PR . Long‐term consequences of benzodiazepine‐induced neurological dysfunction: a survey. PLoS One. 2023;18(6):e0285584. 37384788 10.1371/journal.pone.0285584 PMC10309976

[32] Reid Finlayson AJ , Macoubrie J , Huff C , Foster DE , Martin PR . Experiences with benzodiazepine use, tapering, and discontinuation: an Internet survey. Ther Adv Psychopharmacol. 2022;12:204512532210823. 10.1177/20451253221082386 PMC9047812 35499041

[33] Public Health England. Prescribed Medicines Review: Summary. Public Health England; 2020. https://www.gov.uk/government/publications/prescribed-medicines-review-report/prescribed-medicines-review-summary

[34] National Institute for Health and Care Excellence (NICE) . Medicines Associated with Dependence or Withdrawal Symptoms: Safe Prescribing and Withdrawal (NICE Guideline, No. NG215). NICE; 2022. https://www.nice.org.uk/guidance/ng215/resources/medicines-associated-with-dependence-or-withdrawal-symptoms-safe-prescribing-and-withdrawal-management-for-adults-pdf-66143776880581 35609134

[35] Hirschtritt ME , Olfson M , Kroenke K Balancing the risks and benefits of benzodiazepines. JAMA. 2021;325(4):347‐348. 33416846 10.1001/jama.2020.22106

