# Supplementary material for: ‘We need more support and doctors that understand the process of tapering …’: A content analysis of free‐text responses to a questionnaire on discontinuing long‐term benzodiazepine receptor agonist use
Source: Health Expect. 2024 Jan 7;27(1):e13962. doi: 10.1111/hex.13962 (PMC10771803; doi:10.1111/hex.13962)
Supplement: Supplementary file 1 — Supporting information. [file HEX-27-e13962-s001.docx]

# Appendix S1: Round 1 TDF questionnaire

## General instructions

Thank you for agreeing to complete this questionnaire. Most questions involve ticking the appropriate box. Some questions ask you to write a brief response in the space provided

## Section 1 - Demographics

**Q.1)** What gender do you identify as?

A.) Female B.) Male C.) Non-binary D.) Prefer not to say

**Q.2)** Which age are you?

(Free text box) years

**Q.3)** Which country do you currently reside in?

(Drop down list)

**Q.4)** What is the highest level of education you have completed?

A.) Secondary School/High School B.) Bachelor’s Degree C.) Master’s Degree D.) Other E.) Prefer not to say

**Q.5)** What is your current employment status?

A.) Employed Full-time B.) Employed part-time C.) Unemployed D.) Retired E.) Other (free text box) F.) Prefer not to say

**Q.6)** What is your relationship status?

A.) Single B.) Married C.) Widowed D.) Separated/Divorced E.) Other (free text box) F.) Prefer not to say

**Q.7)** What benzodiazepine(s) or Z-drug(s) are you currently taking?

A.) Alprazolam (Xanax, Gerax) B.) Diazepam (Valium, Anxicalm) C.) Zopiclone (Zimovane) D.) Zopiclone (Stilnoct) E.) Clonazepam (Rivotril) F.) Lorazepam (Ativan) G.) Temazepam (Nortem, Restoril) H.)Other (short free-text box)

**Q.8)** Who prescribed this medicine/these medicines for you?

A.) General Practitioner B.) Hospital doctor C.) Psychiatrist D.) Other (Short answer space)

**Q.9)** Why were you originally prescribed this medication?

A.) Anxiety B.) Insomnia C.) Muscle spasm D.) Other (Short answer space)

**Q.10)** How long have you taken this medication for?

A.) More than 3 months but less than 1 year B.) More than 1 year and less than 5 years C.) More than 5 years and less than 10 years D.)>10 years

**Q.11)** Have you ever attempted stop taking your benzodiazepine or Z-drug medication?

A.) Yes B.) No

**Q.12)** Are you currently tapering from BZRA medication?

A.) Yes B.) No

## Section 2 – Your views and experiences

Please rate your response to each of the statements using the scale below

| Theoretical domain | Questions | 7-point Likert scale |
| --- | --- | --- |
| Knowledge | I know the reasons why I should consider stopping my use of benzodiazepines/Z-drugs | 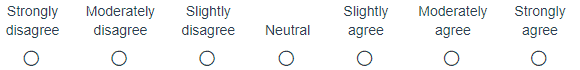 |
|  | I DO NOT know the recommended ways of how to stop using benzodiazepines/Z-drugs | 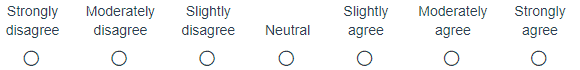 |
|  | I have previously read information about ways of stopping my use of benzodiazepines/Z-drugs | 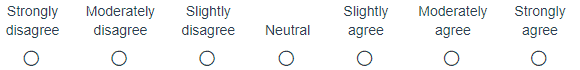 |
| Skills | I don’t have the skills to stop my use of benzodiazepines/Z-drugs | 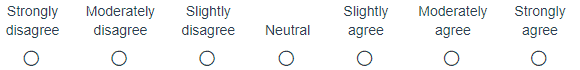 |
|  | I would have to learn new skills to come off my benzodiazepine/Z-drug medication | 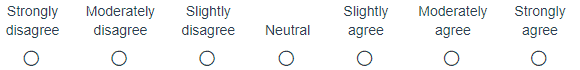 |
|  | I don't have the skills to deal with the symptoms associated with my medical condition without benzodiazepines/Z-drugs | 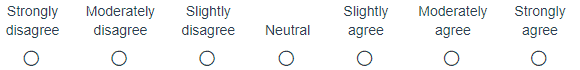 |
| Social/Professional role and identity | My use of benzodiazepines/Z-drugs is now part of who I am | 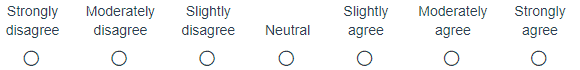 |
|  | I see myself as a benzodiazepine/Z-drug user | 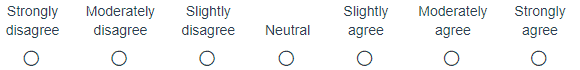 |
|  | It is my doctor's responsibility to ensure I stop my use of benzodiazepines/Z-drugs | 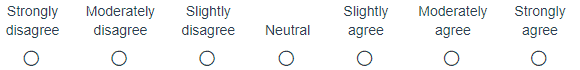 |
| Beliefs about capabilities | I am confident that I can stop my use of benzodiazepines/Z-drugs | 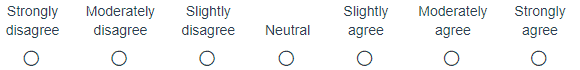 |
|  | Stopping my use of benzodiazepines/Z-drugs would be easy for me | 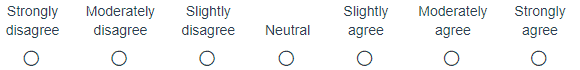 |
|  | I am confident that I will be able to manage the symptoms associated with my medical condition without using benzodiazepines/Z-drugs | 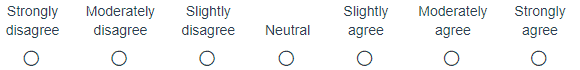 |
| Optimism | I DO NOT feel optimistic about coping with my symptoms without benzodiazepines/Z-drugs | 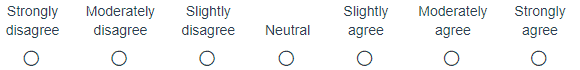 |
|  | I feel it is unrealistic for me to stop my use of benzodiazepines/Z-drugs at some stage in the future | 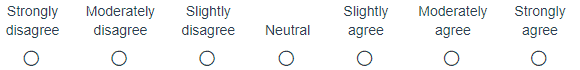 |
|  | I am optimistic that stopping my use of benzodiazepines/Z-drugs will work out well for me | 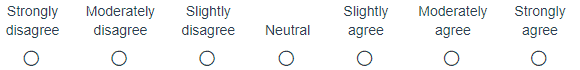 |
| Beliefs about consequences | If I do stop my use of benzodiazepines/Z-drugs, it will benefit me in the short term (e.g. less day time drowsiness, more energy) | 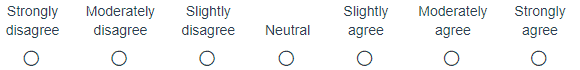 |
|  | If I do stop my use of benzodiazepines/Z-drugs, it will benefit me in the long-term (e.g. sharper thinking, better memory) | 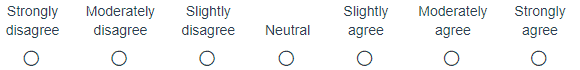 |
|  | I think stopping my use of benzodiazepines/Z-drugs would change my life for the better | 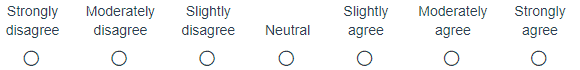 |
| Memory attention and decision processes | I have never considered stopping my use of benzodiazepines/Z-drugs | 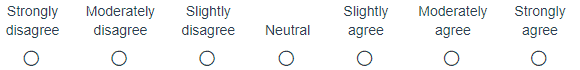 |
|  | I think the benefits of continuing to take benzodiazepines/Z-drugs outweigh the negatives | 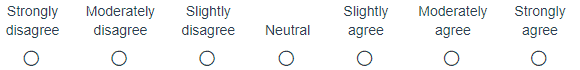 |
|  | Something bad would have to happen to me from taking benzodiazepines/Z-drugs for me to decide to change my current use | 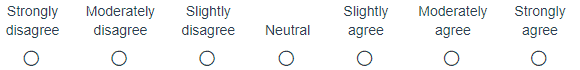 |
| Intentions | I want to stop my use of benzodiazepines/Z-drugs | 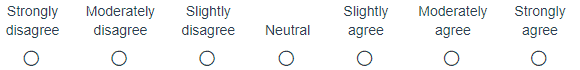 |
|  | I DO NOT have the motivation to stop my use of benzodiazepines/Z-drugs | 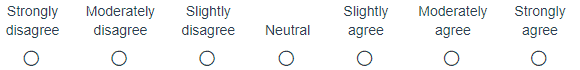 |
|  | I NEVER intended to be taking benzodiazepines/Z-drugs on a long-term basis | 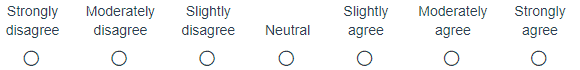 |
| Goals | Stopping my use of benzodiazepines/Z-drugs is NOT my top priority over the next 6-12 months | 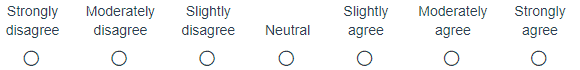 |
|  | I HAVE set myself the goal of stopping my use of benzodiazepines/Z-drugs over the next 6-12 months | 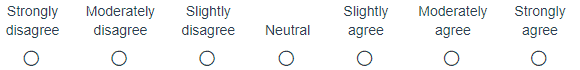 |
|  | My ultimate goal is to one day no longer use benzodiazepines/Z-drugs | 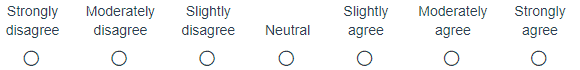 |
| Reinforcement | There is nothing in place that would reward me for trying to stop my use of benzodiazepines/Z-drugs | 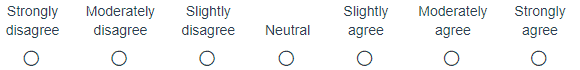 |
|  | Previous failed attempts have had a negative effect on me stopping my use of benzodiazepines/Z-drugs | 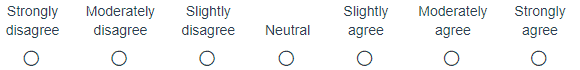 |
|  | The possibility of withdrawal symptoms would discourage me from trying to change my use of benzodiazepines/Z-drugs | 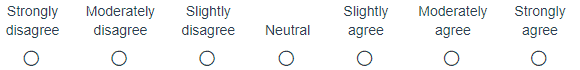 |
| Social influences | I DO NOT have anyone to support me in stopping my use of benzodiazepines/Z-drugs | 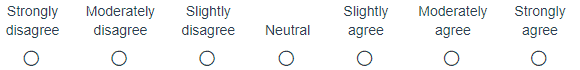 |
|  | I would consider stopping my use of benzodiazepines/Z-drugs if my doctor recommended it | 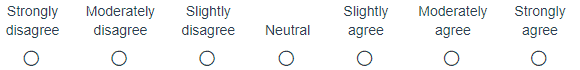 |
|  | My family/friends encourage me to continue taking benzodiazepines/Z-drugs | 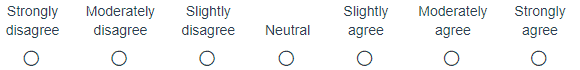 |
| Environmental context and resources | I have access to facilities to help me to stop my use of benzodiazepines/Z-drugs | 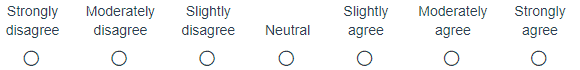 |
|  | There are no services near where I live to support me stopping my use of benzodiazepines/Z-drugs | 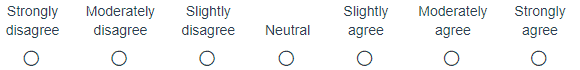 |
|  | I cannot afford the services/supports to help me stop my use of benzodiazepines/Z-drugs | 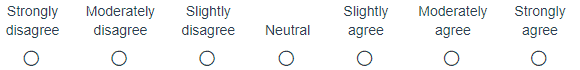 |
| Emotions | Daily life is too stressful for me to stop my use of benzodiazepines/Z-drugs | 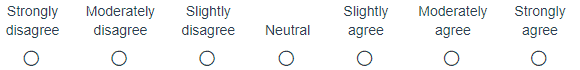 |
|  | I would feel proud for stopping my use of benzodiazepines/Z-drugs | 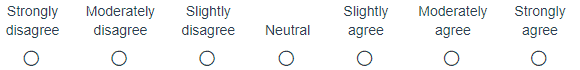 |
|  | When I think about stopping my use of benzodiazepines/Z-drugs, I start to worry | 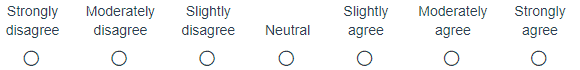 |
| Behavioural regulation | I have a clear plan in mind of how I would stop my use of benzodiazepines/Z-drugs | 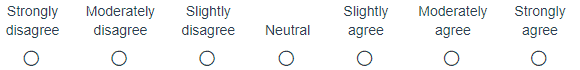 |
|  | I would struggle to stick to a plan to help me stop my use of benzodiazepines/Z-drugs | 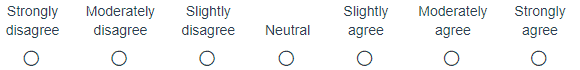 |
|  | I would be able to keep track of my progress in stopping my use of benzodiazepines/Z-drugs | 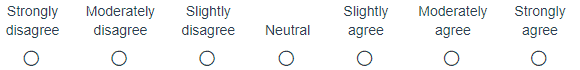 |

Section 3 – Additional comments

1. What is the main barrier preventing you from stopping your use of benzodiazepines/Z-drugs?
2. What would be the main thing that would help you to stop your use of benzodiazepines/Z-drugs?
3. What forms of additional support would you need to help you stop your use of benzodiazepines/Z-drugs?
4. Do you have any additional comments that you would like to add regarding long-term use of benzodiazepines/Z-drugs?

**Thank you for taking the time to complete this questionnaire**
